# Contrast-Enhanced Ultrasound Feasibility in Assessing Carotid Plaque Vulnerability—Narrative Review

**DOI:** 10.3390/jcm12196416

**Published:** 2023-10-09

**Authors:** Ewa Kopyto, Marcin Czeczelewski, Eryk Mikos, Karol Stępniak, Maja Kopyto, Małgorzata Matuszek, Karolina Nieoczym, Adam Czarnecki, Maryla Kuczyńska, Mateusz Cheda, Anna Drelich-Zbroja, Tomasz Jargiełło

**Affiliations:** 1 Students’ Scientific Society, Department of Interventional Radiology and Neuroradiology, Medical University of Lublin, 20-594 Lublin, Poland; ewa.kopyto@gmail.com (E.K.); mikoseryk@gmail.com (E.M.); karol.stepniak3@gmail.com (K.S.); maja.kopyto@gmail.com (M.K.); matuszek.ma@wp.pl (M.M.); karolinanieoczym1@gmail.com (K.N.); adam.czarnecki1234@gmail.com (A.C.); 2 Department of Interventional Radiology and Neuroradiology, Medical University of Lublin, 20-594 Lublin, Poland; maryla.kuczynska@gmail.com (M.K.); mateusz.cheda@gmail.com (M.C.); zbroanna@interia.pl (A.D.-Z.); tomasz.jargiello@umlub.pl (T.J.)

**Keywords:** contrast-enhanced ultrasound, ischemic stroke, vulnerable plaque, atherosclerosis

## Abstract

The risk assessment for carotid atherosclerotic lesions involves not only determining the degree of stenosis but also plaque morphology and its composition. Recently, carotid contrast-enhanced ultrasound (CEUS) has gained importance for evaluating vulnerable plaques. This review explores CEUS’s utility in detecting carotid plaque surface irregularities and ulcerations as well as intraplaque neovascularization and its alignment with histology. Initial indications suggest that CEUS might have the potential to anticipate cerebrovascular incidents. Nevertheless, there is a need for extensive, multicenter prospective studies that explore the relationships between CEUS observations and patient clinical outcomes in cases of carotid atherosclerotic disease.

## 1. Introduction

Cardiovascular diseases (CVDs) persist as the predominant contributors to morbidity and mortality in industrialized countries [1]. Strokes are the third highest cause of death and a common factor leading to disabilities and cognitive deficits [2]. They affect 15 million people worldwide per year; 1/3 of these people are physically challenged for the rest of their lives and 1/3 die [3]. Atherosclerotic plaques within the carotid arteries are a fundamental cause of strokes and transient ischemic attacks (TIAs) while also serving as an indicator for the existence of systemic atherosclerosis [4].

Atherosclerosis is a chronic inflammatory, metabolic, and multifocal process that affects the intima of medium and large arteries [5,6]. Inside the arteries, the bifurcations are particularly susceptible to the formation of atherosclerotic plaques [7]. In this location, hemodynamic stress and turbulent blood flow lead to prolonged damage to endothelial cells [8].

Endothelial dysfunction, the migration and proliferation of macrophages, smooth muscle cells (SMCs), lymphocytes and neutrophils, localized oxidative stress, lipid accumulation, extracellular matrix synthesis, and neovascularization within atherosclerotic plaques collectively contribute to the development of atherosclerosis [9,10]. The disease has a subclinical phase lasting numerous years, with a gradual formation of fatty streaks within arterial walls that evolve over time into atherosclerotic plaques [11,12]. Atherosclerosis can be confined to the arteries supplying a single organ system, although multiple vascular regions are typically involved [13,14]. The initiation of atherosclerotic plaque formation commonly occurs early in life and progresses with age [15,16,17]. Nonetheless, the rate of progression is variable and not entirely predictable among individuals [18].

In the past, evaluating carotid artery stenosis exclusively relied on assessing the extent of narrowing within the artery’s passage, which was regarded as the primary indicator for intervention as per global recommendations [19]. However, relying solely on carotid stenosis for a risk assessment has become obsolete [20]. A shift in the paradigm regarding the relationship between atherosclerosis and cerebrovascular accidents has emerged from contemporary research. Certain high-risk plaques possess considerable damage potential such as causing stenosis or occlusions. These plaques can initiate a sequence leading to cerebral microembolisms, potentially causing ischemic strokes and sudden death [21]. These high-risk plaques are termed “unstable” or “vulnerable” due to their potential for adverse outcomes [22,23]. Notably, the 2023 ESVS guidelines indicate that individuals with only a single risk factor, such as carotid stenosis exceeding 80%, are no longer categorized as being at a high risk of a stroke [24].

## 2. Aim of the Review and Search Strategy

The main objectives of this narrative review were to highlight the current findings related to the application of contrast-enhanced ultrasound in evaluating vulnerable carotid plaques and to relate this method to conventional ultrasound and other modalities. A search of articles related to the topic of this paper was conducted via the use of PubMed, Scopus, and Web of Science databases in June 2023. The first identification aimed to search for and identify the papers utilizing CEUS in the examination of carotid atherosclerotic plaques; the search strategy was (CEUS OR contrast enhanced ultrasound) AND (carotid stenosis OR vulnerable plaque). A second identification of the literature was performed using the following search strategy: (carotid stenosis OR vulnerable plaque) AND (computed tomography OR magnetic resonance OR ultrasound OR imaging). There were no restrictions regarding the year of the publication and we only chose the articles written in English.

## 3. Vulnerable Plaques

Comprehensive investigations have revealed that while severe carotid stenosis remains one of the causes of strokes, individuals with non-severe carotid stenosis may also develop ischemic symptoms [25]. In fact, the most acute cardiovascular incidents arise from the rupture or erosion of the vulnerable plaque phenotypes [26]. The emergence of the vulnerable plaque concept is a result of advancements in the understanding of the natural progression of atherosclerosis, which has enabled the identification of distinct traits that are associated with an increased risk of strokes. Vulnerable plaques are characterized by numerous histological characteristics, including a thin fibrous cap, a lipid-rich necrotic core, high macrophage counts, intraplaque neovascularization, and intraplaque hemorrhages [27]. Atherosclerotic plaques have the potential to undergo a gradual transformation into vulnerable plaques that are prone to inducing local ruptures and thrombosis, eventually occluding the affected artery [28].

Neovascularization is a central pathophysiological phenomenon involved in the development of vulnerable atherosclerotic plaques [29,30,31]. It involves the development of new blood vessels within an atheromatous lesion, facilitated by endothelial cells [32]. Hypoxia and inflammatory conditions induce the release of angiogenic and inflammatory agents, which stimulate spontaneous angiogenesis [33]. Studies have indicated that the neovessels primarily originate from a pre-existing vasa vasorum, which constitutes a microvasculature network responsible for supplying arterial walls [34,35]. Neovessels are commonly found in the fibrous cap as well as in the medial and lateral corners of plaques, but their presence at the base is infrequent [36]. The development of new pathological capillaries promotes macrophage infiltration, inflammation, and lipid deposition as well as intraplaque hemorrhages that contribute to a progressive increase in the instability of plaques [37]. A rupture is commonly found in the plaques where the fibrous cap thickness is less than 0.065 mm and the lipid core volume constitutes 40% of the total plaque volume [38]. Also, an intraplaque hemorrhage is considered to be a risk factor for plaque rupture [39,40].

## 4. Need for Early Identification

The screening for the presence of vulnerable carotid plaques is less routine compared with screening for carotid stenosis as it requires imaging techniques capable of thoroughly examining the morphology of atherosclerotic plaques [41,42]. Nonetheless, the early identification of individuals with vulnerable plaques allows early therapy and, therefore, may prevent clinical complications such as TIAs and strokes [43]. The European Society for Vascular Surgery recommended taking into account the morphological characteristics of plaques when determining the suitability of patients for revascularization interventions such as carotid endarterectomy or carotid artery stenting [24].

## 5. Screening for Vulnerable Plaques

The morphological features of unstable carotid plaques can be identified and measured with the use of advanced vascular imaging technologies [44]. Several non-invasive imaging modalities, including ultrasonography (US), computed tomography (CT), high-resolution magnetic resonance imaging (MRI), and nuclear imaging techniques, have been employed to detect these plaque attributes [45]. The goal is to achieve precise risk stratification and offer insights to guide clinical decision-making [46]. The characteristics of ultrasound make it an excellent imaging modality for the screening of patients with carotid atherosclerosis in the pursuit of identifying vulnerable plaques [47,48]. It is cost-efficient, rapid, and widely available, which provides the possibility for frequent re-examination [49]. Moreover, the characteristics of plaque vulnerability, including ulceration and intraplaque neovascularization, can be evaluated with the use of an intravascular ultrasound contrast agent [50].

## 6. B-Mode US with Doppler

Conventional B-mode US with Doppler is frequently used for first-line examinations of patients with recent cerebrovascular events in order to screen for atherosclerotic disease in peripheral arteries [51,52]. In advanced atherosclerosis, plaques may become visible on an ultrasound examination, allowing for a direct baseline morphological characterization [53,54]. A number of visualized plaques as well as the total plaque area or total plaque volume have been reported to be independent predictors of future cardiovascular mortality and coronary events [55,56].

Plaque vulnerability is a factor that should be at the center of interest for every sonographer because it has a direct impact on the patient’s risk of a stroke [57]. The proper assessment of plaque instability should include the thickness of the fibrous cap, the size of the lipid–necrotic nucleus, ascertaining the presence of plaque neovascularization, determining the direction of atherosclerotic plaque remodeling, and ascertaining the presence of ulceration [58,59]. Vulnerability also defines the type of recommended treatment; it should be conservative in the case of stable plaques or interventional when it comes to unstable, ulcerated plaques [60].

The 2019 European Society of Cardiology (ESC) guidelines for the diagnosis and management of chronic coronary syndromes recommend that carotid B-mode US can be performed on patients with a suspicion of chronic coronary syndrome [61]. Carotid US has a similar risk prediction as the coronary calcium score and can be used in the screening of cardiovascular disease (CVD) [62].

## 7. Gray–Weale–Nicolaides Scale

**Figure 1:**
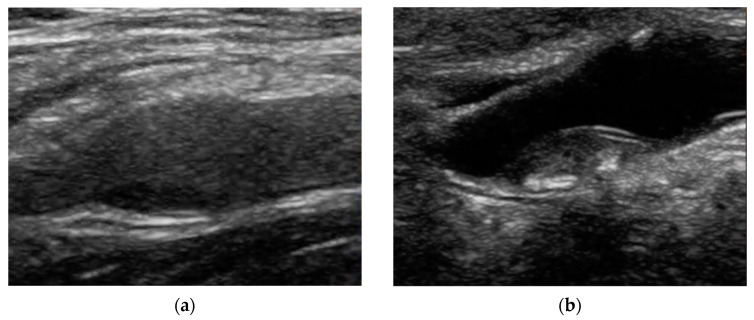
Hypoechoic plaques (types 1 and 2 in Gray–Weale–Nicolaides classification) are associated with intraplaque hemorrhages and lipid accumulation. Type 1 (**a**) is uniformly echolucent and mainly composed of lipid and necrotic components (plaque is marked with an asterisk). Type 2 (**b**) is predominantly echolucent, with small areas of echogenicity caused by calcifications.

Evaluations of the echogenicity of plaques can add information about their presumed lipid, fibrous, or calcium composition, which is crucial for vulnerability assessments [63]. For standardization purposes, the Gray–Weale–Nicolaides scale is applied. It uses a grading system based on echogenicity that classifies atherosclerotic plaques into five types. Type 1 is uniformly echolucent and mostly composed of lipid and necrotic components; type 2 is predominantly echolucent, with small areas of echogenicity caused by calcifications of up to 25% of the plaque volume; type 3 is predominantly echogenic, with small areas of echolucency and calcifications that consist of up to 50% of the plaque volume; type 4 is uniformly echogenic due to calcification of over 50% of the volume; and type 5 consists of plaques that cannot be classified owing to heavy calcification and acoustic shadows [38]. Hypoechoic plaques (types 1 and 2) (Figure 1) are associated with intraplaque hemorrhages and lipid accumulation, whereas hyperechoic homogeneous plaques are predominantly fibrous or calcified in nature [64]. In type 1 and 2 plaques, the risk of ipsilateral strokes is higher due to a greater tendency of disruption or rupture, whereas types 4 and 5 are associated with asymptomatic sclerotization of carotid arteries due to the stabilizing role of collagen and calcifications. Nevertheless, calcified intraluminal plaques may occasionally cause ischemia when a calcified material obstructs part of the brain microcirculation [65,66].

## 8. Intima–Media Thickness

Subclinical atherosclerotic disease can be investigated with the use of a carotid intima–media thickness (IMT) measurement [67,68]. It is the quantitative parameter that is calculated by summing the thickness of the two inner layers within the carotid artery; namely, the intimal and medial layers [49,69]. The normal values for adults range between 650 and 900 μm, with an increase of 0–40 μm every year [49]. A large number of studies have shown a correlation between an increase in the IMT complex value among patients with CVD [70]. However, current guidelines from the ESC do not recommend the use of carotid ultrasound IMT assessments for the screening and risk assessment of asymptomatic cardiovascular patients [71].

The IMT assessment is performed at precisely defined points of individual arteries; specifically, in the proximal, intermediate, and distal CCA and ICA and in the CCA bifurcation [72]. The final IMT value is the average of all calculated measurements [73].

## 9. Ulceration

Carotid plaque ulceration is one of the plaque vulnerability constituents and has long been considered a major risk factor for strokes [74]. Plaque ulcerations are the cause of embolisms much more often than ruptures (74% and 40% of cases, respectively). It should be noted that plaque ulceration and its complications are considered to be the cause of approximately 20% of sudden deaths in both cerebrovascular and cardiovascular events [75].

The investigation of plaque ulcers is not an easy task. Muraki’s criteria are a reference point for a sonographer. They define an ulcer as a cavity on a plaque surface, regardless of its size, where echogenicity at a cavity base should be less than that of an adjacent intima–blood border. Due to a swirling pattern of flow in the ulcer region, the bloodstream forms a “yin-yang” sign that can be observed using a B-mode technique or color Doppler [64]. The characteristic feature of an ulcer is its unusual arrangement in relation to the plaque, which is usually extreme and at the edge of the plaque, with the presence of calcifications on the edge of the ulcer [75].

## 10. Doppler US

Doppler is a feature that long ago became inseparable from ultrasonographic examinations. It can provide accurate information on blood-flow velocities, which is crucial in stenosis assessments. As the flow volume through the vessel is constant, the velocity of the flow is fastest at the stenotic segment. When it comes to carotid arteries, the consensus from 2003 states that a peak velocity starts to increase to above 125 cm/sec in a situation when stenosis rises to over 50% of the vessel lumen. In a 70% stenosis, the mentioned value is doubled. Thus, blood-flow velocity is a marker of segmental stenosis and systemic atherosclerosis [76].

The limitation of this technique is noticeable when the calcified wall of the carotid artery makes it difficult to obtain a proper sonic window. Stenosis can occur on short fragments; thus, the visualization of a vessel for its full length is crucial [76].

In addition, Doppler US can also investigate arterial stiffness, which is one of the structural changes that represent independent predictors of future cardiovascular diseases and susceptibility of arteries to the atherosclerosis process [77]. Arterial stiffness can be measured through the aortic pulse wave velocity (PWV). It is calculated as the distance traveled by the pulse wave divided by the time taken to travel the distance. An increase in arterial stiffness is associated with an increase in the propagation speed of the pulse wave in the arteries. The PWV is estimated from the common carotid artery to the common femoral artery or brachial–ankle PWV. A number of longitudinal studies have shown that the measurement of the PWV can identify subclinical atherosclerosis as well as predict future cardiovascular events [78].

## 11. Contrast-Enhanced Ultrasound

Carotid plaques initially identified from standard unenhanced US images and examined with Doppler US may be further investigated in the context of their intraplaque neovascularization and plaque surface by performing a CEUS examination [79]. Physicians performing CEUS need to familiarize themselves with the technique’s principles (Table 1). This will enable them to precisely interpret findings and promptly identify any artifacts [80].

CEUS utilizes an ultrasonographic contrast agent, an ultrasound machine for the emission of ultrasound beam sequences interacting with tissue, and the software necessary for image post-processing and analysis. Numerous contrast agents are on the market with slightly different compositions; however, they all share a common structure of a microbubble consisting of an internal gas encapsulated by an external shell composed of either phospholipids or albumin [81]. SonoVue^®^ (Bracco, Milan, Italy) is the most commonly employed contrast agent for vascular assessments [82]. It has received European approval for various applications, including cerebral arteries, peripheral arteries, and the portal vein [83].

SonoVue^®^ consists of 2.5 μm bubbles of sulfur hexafluoride gas encapsulated in a monolayer of phospholipids. The phospholipid shell properties allow the gas to be kept inside, yet remain elastic enough to enable the oscillation of microbubbles that is essential for the generation of a CEUS signal. The size of the bubbles is adjusted to allow their free movement inside the capillary bed, but restricts them from passing through the endothelium. Therefore, the SonoVue^®^ contrast agent is exclusively intravascular, which is the basis of its vascular applications [84]. Although this contrast agent is intravenously administered, its route of excretion differs from typical CT or MRI contrast agents. The microbubbles decompose and then the phospholipids are metabolized by the liver while non-toxic sulfur hexafluoride is exhaled by the respiratory system. This property allows SonoVue^®^ to be administered to patients with renal failure, which is a contradiction for other contrast agents [85].

In the context of image acquisition with ultrasonographic contrast agents, the key property is the mechanical index (MI). The MI indicates the insonation power of the ultrasound beam and is defined as the ratio of peak negative pressure to the square root of the US frequency. With ultrasound techniques such as B-mode or Doppler, the MI is usually over 1.6. In contrast to enhanced ultrasonography, the MI value is decreased to values ranging from 0.08 to 0.2 for vascular clinical applications. A low-energy ultrasound beam affects the microbubbles present in the vessels, making them contract and expand in a pattern called oscillation. The contrast agent not only reflects the baseline frequency, but also generates harmonic frequencies as the expansion of microbubbles is stronger than the contraction. Eventually, the bubbles are ruptured by the ultrasound beam, but the area of interest can be observed in real time for a period of a few minutes. The appropriate emission of ultrasound pulses allows for the selective visualization of contrast agents and suppresses the returning signal from static tissue. The results of the CEUS exanimation are displayed on the ultrasound machine screen, juxtaposing a contrast-enhanced image with a low-MI grayscale image for improved orientation [86,87].

## 12. CEUS Assessment of Intraplaque Neovascularization

Intraplaque neovascularization—the accumulation of leaky and fragile capillaries—is involved in the pathogenesis of vulnerable plaques. The pathological vessels destabilize atherosclerotic plaques, making them susceptible to hemorrhaging within the plaque, which can lead to rupture [88]. As the diameter and flexibility of the contrast microbubbles are similar to red blood cells and as they do not leave the lumen of the vessel, the contrast enhancement of the plaque reflects its microvasculature [89]. Later investigations have demonstrated a robust correlation between CEUS enhancement and the histological vascular density of carotid plaques [90,91,92,93].

**Figure 2:**
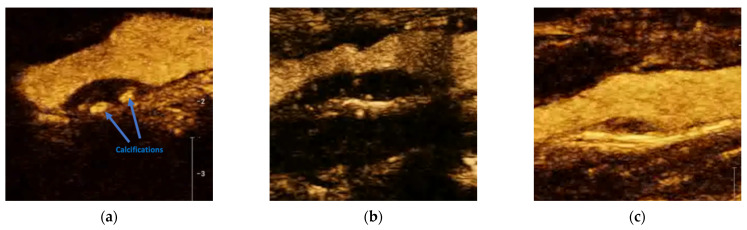
The figure illustrates the qualitative grading system used to assess intraplaque neovascularization within carotid plaques. Grade 1 (**a**) is given to carotid plaques that show no moving microbubbles within the plaque or if there are any, they are limited to the nearby adventitial layer. It is important differentiate artifacts induced by calcification with contrast enhancement (blue arrows). Grade 2 (**b**): there is moderate visibility of moving bubbles within the plaque, mostly at the adventitial side or plaque shoulder. Grade 3 (**c**): there is a clear and visible presence of bubbles moving towards the plaque core or diffused enhancement.

Frequently, intraplaque neovascularization is assessed using a visual-based 3-point grading system (Figure 2) [48,94]. Grade 1 indicates the absence of moving bubbles within the plaque or microbubbles confined to the adventitial layer (no observable microbubbles). Grade 2 denotes a moderate visual presence of bubbles within the plaque at the adventitial side or plaque shoulder (restricted to moderate microbubbles). Grade 3 represents substantial intraplaque neovascularization, characterized by the distinctly visible movement of bubbles towards the plaque core (extensive presence of microbubbles within the plaque).

Furthermore, CEUS can not only evaluate intraplaque neovascularization, but also forecast other histological features of plaque vulnerability. Recent studies correlated contrast enhancement with overall plaque immunohistological vulnerability, including the presence of inflammatory cells, fibrous capsule thickness, and size of lipid core [95,96,97]. The researchers found that CEUS exhibited a sensitivity of 94% and a positive predictive value of 87% in diagnosing histologically vulnerable plaques [93].

## 13. CEUS Assessment of Plaque Surfaces

When evaluating carotid arteries, CEUS provides valuable information at the macrovascular level. Irregularities and ulceration on carotid plaque surfaces hold substantial clinical importance. Numerous studies have linked them to neurologic symptoms and strokes [21,51,98]. Although the evaluation of carotid plaque surface morphology is an integral part of atherosclerosis evaluations, the sensitivity of B-mode and Doppler US can be limited [99,100]. The use of a contrast agent allows for the accurate delineation of plaque surfaces that is independent of a slow blood flow or technical artifacts.

**Figure 3:**
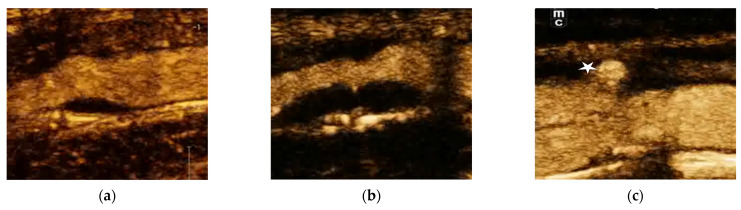
Carotid atherosclerotic plaques exhibit diverse surface morphologies, including smooth, irregular, or ulcerated. In accordance with CEUS imaging, smooth (**a**) denotes a regular plaque surface devoid of significant irregularities. Irregular plaques (**b**) are characterized by surface fluctuations ranging from 0.3 to 0.9 mm. Ulceration (**c**) represents the most severe irregularity, featuring a cavity measuring at least 1 × 1 mm in size (marked with an asterisk).

Carotid atherosclerotic plaques have varying surface morphologies such as smooth, irregular, or ulcerated (Figure 3). For CEUS, the accepted criteria state:

- Smooth refers to a regular surface with no notable irregularities.
- Irregular plaques have fluctuations between 0.3 and 0.9 mm.
- Ulceration is the most severe irregularity, with a cavity of at least 1 × 1 mm [101,102].

Plaque ulceration causes severe endothelial damage, leading to the exposure of plaque’s necrotic core to the circulation, which is strongly related to subsequent ischemic accidents [103]. In several studies comparing conventional color Doppler techniques with CEUS, the latter achieved significantly higher sensitivity in detecting plaque ulceration, reaching up to 88−94% with computed tomography angiography as a reference [100,104]. Apart from plaque surface irregularities, CEUS may be of benefit to the detection of the intraluminal thrombus [105]. When assessing CEUS images, it is crucial to differentiate the hyperechogenic parts of plaques (calcifications) resembling contrast enchantment by cross-checking with low-MI grayscale images [80].

## 14. Quantification of CEUS

Quantitative analysis is currently a vital trend in radiology. It aims to minimize subjectivity and enhance inter-observer consensus. The majority of CEUS studies utilize a qualitative assessment of carotid plaque properties. However, some approaches aim to quantitatively assess the plaque surface and intraplaque enhancement [106,107,108].

For the assessment of intraplaque neovascularization, quantification software analyses the cine loops recorded after contrast agent administration. One of the software packages enabling the automated quantification of intraplaque microvessels is VueBox^®^ v. 7 (Bracco, Milan, Italy), developed by the manufacturer of the SonoVue^®^ agent [109]. Following the administration of the contrast, the enhancement within a designated plaque region of interest is compared with the enhancement within the lumen of the carotid artery on a time–intensity curve. The software allows for the quantification of maximal intraplaque intensity and relative perfused area that can be directly compared between patients.

A quantitative evaluation of carotid plaque surfaces has been proposed as an index obtained by dividing the sum of angular deviations of the plaque surface from a straight line by the plaque’s length [110]; however, this method is not used in solutions available on the market. Quantifying carotid plaque irregularities through CEUS seems both achievable and a promising approach for the identification of vulnerable carotid plaques.

## 15. Clinical Applications

It is valuable to investigate whether CEUS can assess plaque vulnerability and forecast cerebrovascular outcomes in individuals with carotid atherosclerotic disease. Numerous studies have provided indications that CEUS enhancement might correlate with prior or ongoing cerebrovascular events.

Xiong et al. studied the link between CEUS enhancement and a history of transient ischemic attacks (TIAs) or cerebrovascular ischemic strokes. While plaque thickness and ulceration did not significantly differ in symptomatic and asymptomatic patients, statistically significant differences emerged in the enhancement pattern, intensity, and plaque-to-lumen enhancement ratio on CEUS imaging [111]. Likewise, Staub et al. identified a correlation between higher-grade neovascularization in CEUS and increased cardiovascular event frequency [112]. In the same vein, Xu et al. detected significant contrast enhancement differences in cerebral infarction patients [113]. Li et al. reported noteworthy contrast enhancement distinctions in acute ischemic infarction patients [91]. A semi-quantitative CEUS study by Huang et al. revealed stroke-rate disparities between the highest and lowest enhancement grades [114]. Faggioli et al. found heightened CEUS peak enhancement intensity in patients with neurological symptoms [115].

## 16. CEUS versus Other Imaging Methods

Neovascularization and hemorrhaging are histopathological characteristics associated with vulnerable plaques that can result in cerebrovascular accidents [116]. These associations have prompted advancements in imaging tools for the detection of vulnerable carotid plaques (Table 2). CEUS is a recognized and effective imaging technique to assess vascularization [117]. However, it is essential to keep in mind that CEUS shares the limitations of conventional ultrasound. It relies on the operator’s skill and may not provide sufficient information in certain scenarios such as when dealing with large and calcified plaques [118]. Moreover, traditional US imaging is constrained by its ability to assess intravascular events beyond a specific threshold of molecular expression.

High-resolution, multicontrast MRI of the carotid artery also stands out as a tool to identify and quantify different components of atherosclerotic plaques [119]. By employing a combination of diverse MRI sequences such as pre-and post-contrast T1-weighted turbo-spin echo imaging, it becomes possible to discern hemorrhagic regions, calcium deposits, lipid accumulations, and fibrous tissue within carotid plaques [120]. MRI is currently acknowledged as the most valuable imaging method to quantify the carotid plaque burden and non-invasively assess plaque compositions [121]. Its validation, reproducibility, and ability to predict strokes and evaluate treatment outcomes are well-established [121,122].

Furthermore, MRI can be employed to measure plaque inflammation using ultra-small superparamagnetic iron oxide particles (ferumoxtran-10) due to their uptake by macrophages within the plaque [123,124]. While magnetic resonance angiography (MRA) has the advantage of being radiation-free, it comes with limitations such as low spatial and temporal resolutions, resulting in extended scan durations. Additionally, MRA is a complex, costly, and less readily accessible imaging modality when compared with other options [125,126].

CT angiography (CTA) offers the advantage of assessing both extra- and intracranial blood vessels spanning from the aortic arch to the circle of Willis as well as cerebral tissue, all within a single session. In the detection of carotid artery stenosis and irregularities in plaque surfaces, CTA demonstrates sensitivity and specificity on a par with digital subtraction angiography (DSA). It additionally provides insights into the vascular wall and valuable information about plaque compositions [127].

Modern CTA, when combined with specialized segmentation software analyses, can identify the soft-tissue subcomponents of plaques and quantify intraplaque neovascularization [128]. The degree of contrast enhancement appears to correlate with the extent of neovascularization [129]. Studies have shown that multidetector CT angiography outperforms DSA and ultrasound in detecting ulcerations with higher sensitivity and specificity [99]. Nonetheless, a certain level of controversy exists in the literature regarding CTA’s reliability in characterizing soft-tissue components. Some authors have reported lower sensitivity in distinguishing lipid cores and intraplaque hemorrhages when dealing with small lesions [130].

## 17. Conclusions

In conclusion, CEUS is a significant tool when evaluating the sonographic indicators of carotid plaque vulnerability, portraying surface irregularities, ulceration, intraplaque neovascularization, and adventitial vasa vasorum development (Table 3). CEUS-detected intraplaque neovascularization closely aligns with histological capillary density, indicating vulnerability. Preliminary evidence suggests CEUS could directly predict the high-risk plaque histologic grade. CEUS correlates with past cerebrovascular events and shows potential for forecasting future cerebrovascular and cardiovascular incidents. Despite progress, CEUS studies often lack a prospective design. Larger multicenter studies are needed to further establish CEUS’s role in carotid atherosclerotic disease assessments.

## Figures and Tables

**Table 1:** Carotid contrast-enhanced ultrasound (CEUS) workflow and technical details of the procedure.

Carotid CEUS Workflow	Reference/Setting
Ultrasound machine setup	Ultrasound machine with pulse-inversion technique MI < 0.2 Linear array probe with frequency range of 3–11 MHz
Ultrasonographic contrast agent administration	2.4 mL SonoVue^®^ flushed with 5–10 mL of 0.9% saline Bolus injection from antecubital fossa or central line Large lumen cannula (<20 gauge) to avoid microbubble damage
Image acquisition	Contrast agent usually appears in the carotid arteries within 20–30 s Cine loop of 280–360 s is recorded
Image analysis	Qualitative assessment of recorded cine loops Post-processing analysis with quantification

**Table 2:** Application scenarios, advantages, and limitations of the various non-invasive techniques used for plaque vulnerability examinations.

Imaging Technique	Application Scenarios	Advantages	Limitations
CEUS	Contrast agent indicates regions with high neovascularization Good visualization of plaque surface and ulceration	Non-invasive Radiation-free Good availability Limited cost	Operator dependency Variability Resolution
CT	High resolution allows for examination of plaque density and ulceration Most effective technique for detection of calcification	Non-invasive High resolution Reproducibility	Radiation Contrast agents Artifacts present due to calcification
MRI	Good differentiation between fibrous cap and necrotic lumen Intraplaque hemorrhage detection	Non-invasive Radiation-free High resolution Reproducibility	Gadolinium contrast is often needed Costs Time Availability

**Table 3:** Indicators of carotid plaque vulnerability detected by carotid contrast-enhanced ultrasound (CEUS).

Marker	Classification	Details
Plaque surface	Visual-based qualitative system	Smooth plaque surface Plaque surface irregularities (0.3 and 0.9 mm) Plaque ulceration (>1 × 1 mm)
	Surface irregularity index	Index obtained by dividing the sum of angular deviations of the plaque surface from the straight line by the length of the plaque
Intraplaque neovascularization	Visual-based qualitative system	Grade 1: no vascularization Grade 2: moderate vascularization Grade 3: extensive vascularization
	Quantitative assessment with dedicated software, e.g., VueBox	Plaque region of interest compared with the lumen of the carotid artery

## Data Availability

Not applicable.
